# Injectable Gelatin–Palmitoyl–GDPH Hydrogels as Bioinks for Future Cutaneous Regeneration: Physicochemical Characterization and Cytotoxicity Assessment

**DOI:** 10.3390/polym17010041

**Published:** 2024-12-27

**Authors:** Aifa Asyhira Khairul Nizam, Nur Izzah Md Fadilah, Haslina Ahmad, Manira Maarof, Mh Busra Fauzi

**Affiliations:** 1 Department of Tissue Engineering and Regenerative Medicine, Faculty of Medicine, Universiti Kebangsaan Malaysia, Cheras, Kuala Lumpur 56000, Malaysia; p138495@siswa.ukm.edu.my (A.A.K.N.); izzahfadilah@ukm.edu.my (N.I.M.F.); manira@ppukm.edu.my (M.M.); 2 Advance Bioactive Materials-Cells UKM Research Group, Universiti Kebangsaan Malaysia, Bangi 43600, Selangor, Malaysia; 3 Department of Chemistry, Faculty of Science, Universiti Putra Malaysia (UPM), Serdang 43400, Selangor, Malaysia; haslina_ahmad@upm.edu.my; 4 Integrated Chemical Biophysics Research, Universiti Putra Malaysia (UPM), Serdang 43400, Selangor, Malaysia

**Keywords:** injectable hydrogel, bioinks, 3D bioprinting, gelatin, elastin, palmitoyl–GDPH, skin, wound healing

## Abstract

Tissue engineering and regenerative medicine have made significant breakthroughs in creating complex three-dimensional (3D) constructs that mimic human tissues. This progress is largely driven by the development of hydrogels, which enable the precise arrangement of biomaterials and cells to form structures resembling native tissues. Gelatin-based bioinks are widely used in wound healing due to their excellent biocompatibility, biodegradability, non-toxicity, and ability to accelerate extracellular matrix formation. However, the role of a novel fatty acid conjugated tetrapeptide, palmitic acid–glycine–aspartic acid–proline–histidine (palmitoyl–GDPH), in enhancing hydrogel performance with human dermal fibroblasts (HDFs) concerning cell survival, proliferation, growth, and metabolism remains poorly understood. This study fabricated gelatin–palmitoyl–GDPH hydrogels at various concentrations (GE_GNP_ELS_PAL12.5 and GE_GNP_ELS_PAL25) using an injectable method and preliminary extrusion-based 3D bioprinting at 24 °C. Physicochemical characterization revealed superior water absorption, biocompatibility, and stability, aligning with optimal wound-healing criteria. In vitro cytotoxicity assays demonstrated >90% cell viability of HDFs cultured on these scaffolds for five days. These results highlight their ability to promote cell survival, proliferation, and adhesion, establishing them as strong contenders for wound healing. This study underscores the potential of gelatin–palmitoyl–GDPH hydrogels as effective bioinks for 3D bioprinting, offering a promising platform for skin tissue engineering and regenerative medicine.

## 1. Introduction

Over recent decades, tissue engineering has revolutionized therapeutic approaches to wound healing by introducing advanced biomaterials capable of mimicking the native extracellular matrix (ECM). Among these innovations, injectable hydrogels have emerged as a highly versatile platform, offering minimally invasive delivery of therapeutic agents and cells directly to injury sites. These hydrogels, which transition from liquid to gel under physiological conditions, create an optimal microenvironment for cellular attachment, proliferation, and tissue regeneration, overcoming the limitations of conventional care methods [1,2]. It is widely believed that cells encapsulated within hydrogels use focal adhesion to sense and respond to their biomechanical environments [3]. Hydrogels are three-dimensional (3D) polymeric networks primarily composed of water, enabling them to replicate the moisture-rich environment of natural tissues. Their diverse chemical and physical properties confer unique advantages for biomedical research, including high flexibility for fabrication tailored to specific applications [4]. Moreover, their ability to retain moisture minimizes tissue necrosis and accelerates epithelial regeneration. The primary advantage of hydrogel structures lies in their capacity to mimic the extracellular matrix (ECM), enabling the controlled release of functional biomolecules while preserving the activity of embedded cells and bioactive substances [1]. These distinctive properties position hydrogels as promising candidates for advanced biomedical applications, particularly in tissue engineering and regenerative medicine.

Recent advancements in conventional fabrication techniques have laid the foundation for improved 3D-bioprinting methods, which have transformed tissue engineering by enabling the precise layer-by-layer construction of biomimetic structures closely resembling native tissues. 3D bioprinting employs computer-aided design (CAD) data to control the spatial arrangement of bioinks. Bioprinting approaches include several key methods: extrusion-based bioprinting, which constructs 3D biomatrices; inkjet bioprinting, which delivers precise droplets of low-viscosity bioinks; laser-assisted bioprinting, which achieves high-resolution deposition of materials in solid or liquid phases; acoustic bioprinting, which builds 3D structures using surface acoustic wave technology; magnetic bioprinting, which utilizes magnetic fields to manipulate and position magnetically responsive bioinks; and stereolithography, a technique that fabricates 3D patterned scaffolds with micro- and nano-architectures [5,6,7,8]. Bioinks, as viscous liquids, form continuous strands composed of cells embedded in gellable materials. These bioinks are deposited layer-by-layer to construct complex 3D structures precisely [9,10]. This intricate process aligns closely with the principles of tissue engineering, aiming to replicate natural cellular microenvironments. Beyond facilitating cellular differentiation, bioinks serve as therapeutic tools that foster optimal environments for cell growth and interaction, both essential for the successful development and regeneration of organs. This approach not only enhances the functional capabilities of tissue engineering strategies but also bridges the gap between natural biological processes and synthetic fabrication methods.

Advanced biomaterials have been extensively studied as substitutes for biological tissues, particularly skin, to replace damaged parts of the human body [11]. Skin substitutes are heterogeneous biomaterials designed to replace the extracellular matrix and promote wound healing. They offer several advantages, including wide accessibility, durability, antigen-free composition, and resistance to infection [12]. A broad range of biomaterials is available for skin wound healing, encompassing both natural and synthetic types. Natural biomaterials, which are biological products derived from bacterial, fungal, plant, or animal sources, can be classified into proteins, polysaccharides, or macromolecules that combine both. In contrast, synthetic biomaterials are made from organic and inorganic polymers [13]. These materials often exhibit important characteristics such as biocompatibility, structural similarity to natural tissues, mechanical strength, tunable degradation rates, and the ability to facilitate cellular interactions. Such physicochemical properties are critical for their application in human skin and other organs. Recently, the integration of bioactive compounds into biomaterials has gained significant attention due to their therapeutic effects, including enhanced cell proliferation and migration, collagen synthesis, and antimicrobial activity. These properties are particularly valuable in skin wound healing. Bioactive peptides such as elastin-like polypeptide (ELP), –Ser-Ile-Lys-Val-Ala-Val (SIKVAV), and palmitic acid–glycine–aspartic acid–proline–histidine (palmitoyl–GDPH) offer multifaceted biological effects that highlight their potential to revolutionize tissue engineering, regenerative medicine, and dermatology [14,15,16,17].

Among the biomaterials attracting significant attention, collagen and its derivatives are noteworthy due to their biocompatibility with bioactive compounds. Collagen, a primary structural protein in the extracellular matrix, plays a vital role in wound healing and tissue regeneration [18]. The triple-helix structure of collagen is broken down into single strands in its partially hydrolyzed form, known as gelatin. Gelatin, a denatured form of collagen, is produced by controlling the hydrolysis of fibrous collagen and retains nearly identical chemical properties to its precursor. This natural biomaterial can be categorized based on its extraction process into type A (positively charged) and type B (negatively charged) gelatin. Techniques for gelatin extraction and its sources are similarly varied, as it is a collagen derivative [11]. Gelatin demonstrates high fidelity, adaptability, biocompatibility, degradability, low viscosity, and excellent water retention and rheological properties, making it a preferred material for modern bioprinting applications [19,20]. Additionally, the presence of arginine–glycine–aspartate (RGD) motifs within its structure facilitates cell adhesion, proliferation, and migration [21]. Moreover, gelatin is non-toxic, more soluble in most solvents, and has a lower antigenicity compared to collagen [22,23]. These attributes have made gelatin highly sought after in the global market, with a demand of 412.7 kilotons in 2015, primarily sourced from pigskin [24]. Gelatin also serves as a hemostatic agent, initiating the wound-healing process by absorbing exudates and creating an optimal environment for the inflammatory phase, while providing a porous scaffold to promote fibroblast migration to the injury site [25]. In this study, gelatin and polyvinyl-alcohol (PVA) were used as bioinks in extrusion-based 3D bioprinting. The hypothesis driving this research is that both PVA and gelatin facilitate physiological processes that enhance biocompatibility, exhibit excellent physicochemical properties, and develop biostable rheological properties [26]. The combination of gelatin and PVA has garnered considerable interest as a potential bioink for wound-repair applications [5]. This underscores the notion that a single polymer may not always suffice to meet all the specifications for tissue regeneration.

Thus, combining natural and synthetic biomaterials in composite bioscaffolds can facilitate the development of skin substitutes tailored to factors such as fabrication techniques, wound size and shape, patient age, and burn severity, ensuring that all medical requirements are met. Additionally, the incorporation of crosslinkers and bioactive compounds can enhance mechanical properties and biocompatibility. A novel bioactive peptide was developed for this purpose. This therapeutic peptide was synthesized via solid-phase peptide synthesis (SPPS) and formed palmitic acid conjugated to glycine–aspartic acid–proline–histidine (palmitoyl–GDPH) [17]. The palmitoyl–GDPH sequence is derived from the well-known RIGIN sequence (arginine–glutamine–isoleucine–asparagine), also known as palmitoyl tetrapeptide-7, and has been widely studied in skin care for its role in reducing facial wrinkles [27]. Modifications to the RIGIN sequence (palmitoyl-GQPR) replacing glutamine (Q) and arginine (R) with aspartic acid (D) and histidine (H), respectively, aim to improve peptide activity, particularly in promoting keratin function facilitated by D and H, a key factor in skin regeneration and skin barrier function. The N-terminal of the peptide is conjugated to palmitic acid, while the C-terminal is amidated to optimize stability and bioactivity for skin treatments [28]. This novel small peptide is water-soluble, has a pH of 3.6 when dissolved in water, and exhibits anti-inflammatory properties, effectively inhibiting collagenase and promoting wound healing [28]. Moreover, palmitoyl–GDPH promotes cell migration, is biocompatible, non-cytotoxic, and enhances human skin fibroblast growth [28], likely by increasing extracellular matrix proteins [29]. In vivo studies have demonstrated its ability to accelerate wound closure without significant toxicity or systemic side effects in the liver, kidney, or pancreas [17].

Elastin, another natural biomaterial, improves the elasticity and flexibility of skin. Sources of elastin include chicken skin (22% protein content), rat and rabbit lungs, pig and human aorta, and bovine ligamentum nuchae [30,31,32], used in pharmaceutical, medical, and tissue engineering applications. In tissue engineering, elastin may be incorporated as insoluble or hydrolyzed fibers or as a copolymer with additional biopolymers [33]. Elastin has shown potential as an antioxidative peptide with good biocompatibility and non-toxic effects [30]. Notably, elastin extracted from boiled skin and processed into water-soluble elastin powder offers a promising alternative for use in tissue engineering [30]. In addition to elastin, genipin, a natural crosslinking agent derived from *Gardenia jasminoides* and *Genipa americana*, has been extensively documented for its excellent biocompatibility, biodegradability, and stability as a crosslinker in biomaterials [34,35,36]. Genipin has shown to be 10,000-fold less toxic than glutaraldehyde and promotes fibroblast adhesion and proliferation, whereby cells multiplied more than 5000-fold during cytotoxicity assay, making it an attractive option for tissue engineering applications [23]. Previous studies have shown that gelatin crosslinked with genipin is biocompatible with and non-toxic to human skin fibroblasts [37], advancing the clinical potential of gelatin-based products in regenerative medicine and wound healing.

**Figure 1:**
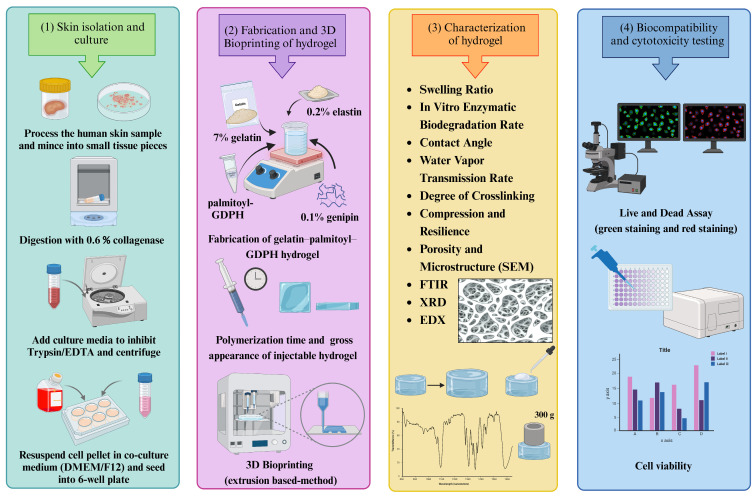
The study flow of the injectable gelatin–palmitoyl–GDPH hydrogel. Images were created by using BioRender.com.

Building on this foundation, this study developed an injectable gelatin–palmitoyl–GDPH hydrogel crosslinked with genipin and incorporated with water-soluble elastin powder as a potential biomatrix for chronic wound healing. The physicochemical, mechanical, and cytotoxic properties were thoroughly assessed. The results suggest that the inclusion of elastin and genipin enhances the hydrogels’ mechanical strength and biological activity, positioning it as an ideal candidate for 3D-bioprinting applications. Furthermore, the hydrogel exhibited promising potential to stimulate cell proliferation in chronic wound regions, making it a suitable bioink for wound care management and tissue regeneration. The study flow is illustrated in Figure 1.

## 2. Materials and Methods

This study design received approval from the Universiti Kebangsaan Malaysia Research Ethics Committee (Grant Code: FF-2024-370 and Ethics Approval Code: JEP-2024-703).

### 2.1. Materials

Commercial gelatin (GE) powder (Nitta-Gelatin Ltd., Yao City, Osaka, Japan) was used to fabricate the gelatin-based hydrogel. The natural crosslinker genipin was purchased from FUJIFILM Wako Pure Chemical Corporation, Chuo-ku, Osaka, Japan, and dissolved in 70% ethanol (MERCK, Darmstadt, Germany). The palmitoyl–GDPH peptide used in this study was sourced from GL Biochem Shanghai Ltd., Shanghai, China. Phosphate-buffered saline (PBS) powder from FUJIFILM Wako Pure Chemical Corporation, Chuo-ku, Osaka, Japan, was used as a buffer for the swelling ratio test, while ninhydrin (Sigma Aldrich, Saint Louis, MO, USA) was used to determine the degree of crosslinking of the hydrogel. A 0.0006% collagenase type I solution (Worthington, Lakewood, NJ, USA) was used to assess the in vitro enzymatic biodegradation rate. Distilled water was used for several characterization tests. Additionally, an in vitro biocompatibility test was conducted using the LIVE/DEAD cytotoxicity test for mammalian cells (Thermo Fisher Scientific, Waltham, MA, USA) on cultured primary human dermal fibroblast (HDF) cells.

### 2.2. Preparation and Fabrication of Hydrogel

Commercial gelatin (GE) powder (Nitta-Gelatin Ltd., Yao City, Osaka, Japan), with a molecular weight (Mw) of 100 kDa, was dissolved in distilled water (dH_2_O) at a concentration of 7% (*w*/*v*) at 40 °C. Genipin (GNP) (FUJIFILM Wako Pure Chemical Corporation, Chuo-ku, Osaka, Japan) solution was prepared to 0.1% (*w*/*v*) with 70% ethanol (EtOH) (MERCK, Darmstadt, Germany), which was then mixed with the gelatin solution under continuous stirring at 40 °C for 30 min to ensure homogeneity. A water-soluble elastin (ELS) solution (0.2% *w*/*v)* from Kamaruzaman et al. (2022) was added, followed by palmitoyl–GDPH (12.5 µg/mL and 25.0 µg/mL; purity ≥ 98%, Mw 662.82 Da; GL Biochem Shanghai Ltd., Shanghai, China). The final formulations were prepared by varying the palmitoyl–GDPH concentrations and incorporating ELS, yielding GE_GNP_ELS_PAL12.5, GE_GNP_ELS_PAL25, and GE_GNP_ELS, with GE_GNP as the control. Polymerization times were evaluated using the inverted tube test at ±23 °C. For printed hydrogels, the bioinks were loaded into syringes and extruded using a 3D bioprinter (Biogens XI, 3D GENS, Shah Alam, Selangor, Malaysia) at 23 ± 2 °C.

### 2.3. Evaluation of Gross Appearance

The gross appearance of the fabricated hydrogels was captured using a digital camera (Nikon, Tokyo, Japan), including top and lateral views. Additionally, a 3D bioprinter (BiogensX1) was used to print the hydrogel, and the overall gross appearance was evaluated.

### 2.4. Swelling Ratio

The swelling properties of the hydrogels were assessed to evaluate their capacity to absorb wound exudates, following a protocol adapted from previous studies [37]. The hydrogels were first freeze-dried and weighed to record the initial dry weight (Wi). They were then immersed in phosphate-buffered saline (PBS, pH 7.4) for 6 h at room temperature. After removing excess PBS from the hydrogel surface with filter paper, the final weight (Wf) was observed. The swelling ratio was calculated using the following formula:

$$Swelling\;Ratio\left(\%\right)=\frac{Wf-Wi}{Wi}\times 100$$

where Wf represents the hydrogels’ weight after immersion, and Wi represents the hydrogels’ weight before immersion.

### 2.5. In Vitro Enzymatic Biodegradation

Enzymatic biodegradation was performed to assess the hydrogel’s biodegradability and interactions with physiological enzymes, following a method from a previous study [5]. The hydrogels were first weighed to obtain the initial weight (Wa) before being immersed in a 24-well plate containing 0.0006% (*w*/*v*) collagenase type I (Worthington, Lakewood, NJ, USA). The samples were incubated at 37 °C for 6 h to allow enzymatic degradation. After incubation, the hydrogels were thoroughly rinsed with distilled water to remove residual salts and reweighed to obtain the final weight (Wb). The percentage of weight loss, indicating biodegradability, was calculated using the following formula:

$$Weight\;Loss\left(\%\right)=\frac{Wa-Wb}{Wa}\times 100$$

where Wb represents the final weight, and Wa represents the initial weight.

### 2.6. Contact Angle

The contact angle test was performed to evaluate the surface properties of the polymerized hydrogels, specifically their hydrophilicity. First, 10 µL of distilled water was carefully dropped on the surface of the hydrogels, and images were captured using a digital camera. The captured images were analyzed using ImageJ Software (National Institute of Health, V1.5, Bethesda, MA, MD, USA).

### 2.7. Water Vapor Transmission Rate

The water vapor transmission rate (WVTR) of the hydrogels was determined according to the American Society for Testing and Materials (ASTM) standards [38,39] to evaluate their capacity for water evaporation and gas exchange, which are critical for wound healing. Hydrogels were placed over a cylinder containing 10 mL of distilled water, and the setup was incubated at 37 °C with 5% CO_2_. The WVTR was calculated using the following equation:

$$WVTR=\frac{\left(Wi-Wf\right)}{A\times Time}$$

where Wi represents the initial weight, Wf represents the final weight, and A is the cylinder bottle’s surface area.

### 2.8. Mechanical Properties Analysis

The mechanical properties of the hydrogels were evaluated using a modified compression test [38,39] to assess their strength and suitability for tissue engineering applications. Polymerized hydrogel samples, with a diameter of 2 cm and a height of 5 mm, were subjected to compression at room temperature. The compressive modulus (E) was calculated using the following formula [40]:

$$E=\frac{\sigma }{\varepsilon }$$

where σ represents compressive force per unit (stress), and ε represents changes in volume per unit volume (strain).

### 2.9. Resilience

Resilience was assessed by measuring the ability of the scaffolds to recover after compression, following a method adapted from [39]. A 300 g metal load was applied to the scaffolds for 5 min. Pre- and post-compression images of the hydrogels were captured using a digital camera with a scale, and thicknesses were analyzed using the ImageJ software version 1.54k (NIH, Bethesda, MD, USA). The following equation was utilized to determine the percentage of resilience (R):

$$R\left(\%\right)=\frac{Ai-Ac}{Af}\times 100$$

where Ai represents the thickness area prior to compression, Ac represents the thickness area when being compressed, and Af represents the thickness area after compression.

### 2.10. Degree of Crosslinking

The ninhydrin assay (Sigma Aldrich, Saint Louis, MO, USA) was employed to evaluate the degree of crosslinking, which quantifies free amino groups in hydrogels [41]. This assay compares the free amino groups in crosslinked hydrogels, which interact with genipin, to those in non-crosslinked hydrogels. The assay was conducted in dark conditions because of the light-sensitive nature of ninhydrin. Glycine standard curves were prepared by serial dilutions at concentrations of 0.006, 0.0125, 0.025, 0.05, and 0.1 mg/mL. Hydrogels were lyophilized for 24 h and weighed to 10 mg per scaffold. The optical absorbance at 570 nm was measured using a spectrophotometer (BioTek, PowerWave XS, Highland Park, Winooski, Vermont, IL, USA) after boiling the samples for 2 min at 100 °C. The degree of crosslinking was calculated using the following formula:

$$Degree\;of\;Crosslinking=\frac{Anc-Ac}{Anc}\times 100$$

where Anc represents non-crosslinked hydrogels, and Ac represents crosslinked hydrogels’ absorbance.

### 2.11. Porosity Measurement

Two distinct approaches were used to determine the porosity percentage of the freeze-dried hydrogels, as detailed below.

#### 2.11.1. Scanning Electron Microscopy (SEM)

The scaffolds were freeze-dried overnight to produce lyophilized hydrogels and then coated with a nanogold layer using ion sputtering. The hydrogel network structure was observed using a field-emission scanning electron microscopy (FESEM) (Supra 55VP model, Jena, Germany), following an established study [39]. This analysis is crucial for characterizing the microstructure, providing high-resolution images of the hydrogel surface and internal architecture, including pore size, distribution, and connectivity.

#### 2.11.2. Liquid Displacement Method

Freeze-dried hydrogels were dehydrated by immersion in 99.5% ethanol. Ethanol, a non-polar liquid, permeated the matrix and filled all pores without reacting with polymeric fibers. Scaffold weights were recorded before and after ethanol submersion in accordance with prior research [42]. This method is vital for characterizing the porosity and structural integrity of the hydrogels. To calculate the porosity percentage of the scaffold, the following equation was used:

$$Porosity\left(\%\right)=\frac{Wf-Wi}{pV}\times 100$$

where Wi represents the initial weight of the scaffold, Wf represents the final weight of the scaffold, V represents the volume of the scaffold, and ρ is the density of ethanol (0.789 g/m^3^).

### 2.12. Fourier Transform Infrared Characterization of Hydrogel Samples

A Fourier transform infrared spectrometer (FTIR) (PE, Waltham, MD, USA) was utilized to determine functional groups within the hydrogels, covering a wavelength range from 4000 cm^−1^ to 500 cm^−1^. The chemical structure, including alterations by crosslinking or the incorporation of water-soluble elastin and palmitoyl–GDPH peptide, was analyzed by evaluating absorbance peaks. Elemental composition was assessed via energy dispersive X-ray (EDX) analysis using a Phenom Pro X SEM EDX microscope (Phenom, Eindhoven, The Netherlands). Control samples included palmitoyl–GDPH powder, gelatin, genipin, and water-soluble elastin. Additionally, hydrogel crystallinity was evaluated using an X-ray diffractometer (Bruker, D8 Advance, Coventry, UK) operating from 0 to 80 °C and with a diffraction angle of 2θ. The integrated software (Diffrac. Suite EVA, V4.0, Bruker, Coventry, UK) was used for further diffractogram analysis.

### 2.13. Skin Isolation and Culture

Human skin samples were obtained as redundant tissue from three voluntary surgical patients. Primary human dermal fibroblasts (HDFs) were isolated to fabricate a bio-engineered matrix for with chronic wound repair. Skin samples were trimmed into 1 × 3 cm pieces with fat and excess dermal tissue removed, washed with sterile Dulbecco’s phosphate-buffered saline (DPBS), and minced into smaller fragments. The fragments were digested with 0.6% collagenase type I (Worthington-Biochemical Corporation, 730 Vassar Ave Lakewood, NJ, USA) in a shaker incubator at 37 °C for 4–6 h. This was followed by a 10-min trypsinization using 0.05% of trypsin-EDTA (Gibco, Carlsbad, CA, USA). The resulting cell suspension was centrifuged and resuspended in co-culture medium composed of Epilife and F12:DMEM (1:1) (Gibco/BRL, Carlsbad, CA, USA) supplemented with 10% fetal bovine serum (FBS) (Biowest, Bradenton, FL, USA). Cells were seeded at a density of 1 × 10^4^ cells/cm^2^ in a 6-well polystyrene plate and incubated at 37 °C with 5% CO_2_. The culture medium was replaced every 2–3 days, and differential trypsinization was performed once cells reached 70–80% confluency. HDFs were subsequently expanded in a 75 cm^2^ flask with F12:DMEM with 10% FBS.

### 2.14. Live and Dead Assay

The LIVE/DEAD Cytotoxicity Kit (Thermo Fisher Scientific, Waltham, MA, USA) was used to evaluate the biocompatibility of hydrogels with cultured HDFs. Sterile gelatin-palmitoyl–GDPH hydrogels were fabricated in a biosafety cabinet under sterile conditions. Biomaterials including gelatin, genipin, water-soluble elastin, and palmitoyl–GDPH were sterilized under UV light (BioAir Safemate^TM^ EZ Series, Siziano, Pavia, Lombardia 12, Siziano, Italy) at 151 µW/cm^2^ for 20 min. Distilled and deionized water, both autoclaved, were used to dissolve the biomaterials. The sterile hydrogels were placed in a 48-well polystyrene culture plate, polymerized, and seeded with passage-3 HDFs (3.5 × 10^4^) for 24 h [5]. After incubation for 30minutes at 37 °C with a working solution containing 2 mM calcein-AM and 4 mM ethidium homodimer-1 (EthD-1), cytotoxicity was analyzed using a fluorescence microscope (Nikon A1R-A1, Tokyo, Japan) at 100× magnification.

### 2.15. Statistical Analysis

All data were analyzed using GraphPad Prism version 10.0 (GraphPad Software, Inc., San Diego, CA, USA). Statistical comparisons across groups were performed using one-way and two-way analysis of variance (ANOVA). Data are presented as mean ± standard deviation (SD), with statistical significance set at *p*-value < 0.05. All experiments were conducted in triplicates (n = 3).

## 3. Results

### 3.1. Gross Appearance and Polymerization Time

**Figure 2:**
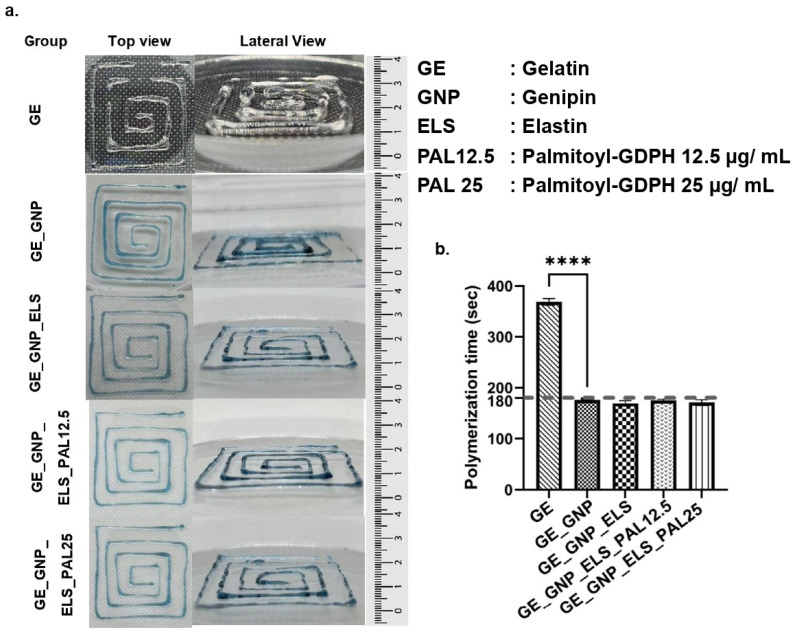
(**a**) Gross appearance with top view and lateral view of all groups of hydrogels and (**b**) polymerization time for all hydrogel groups, where **** indicates *p* < 0.0001.

Genipin (GNP) was successfully used to chemically crosslink gelatin–palmitoyl–GDPH hydrogels, with water-soluble elastin (ELS) effectively incorporated. The process is characterized by a progressive color change from yellowish to greenish and finally to blue during fabrication, indicating the genipin crosslinking reaction. The fully crosslinked hydrogel, with a blue gross appearance, signifies the formation of covalently crosslinked networks as genipin reacts with primary amine groups in gelatin. The blue pigment results from oxygen radical polymerization of genipin and dehydrogenation of intermediate compounds, following a ring-opening reaction triggered by primary amine group attack. This radical reaction represents the final step in a series of crosslinking reactions [43]. In Figure 2a, gelatin hydrogels (GE) appear clear, indicating incomplete crosslinking and the absence of the blue pigment. The observed color transition from yellowish to greenish and then to blue provides visual evidence of crosslinking progression during gelatin–palmitoyl–GDPH hydrogel fabrication, confirming the hydrogels have reached a fully crosslinked state. Gross appearance, including top and lateral views, and polymerization times for each hydrogel formulation (GE_GNP, GE_GNP_ELS, GE_GNP_ELS_PAL12.5, and GE_GNP_ELS_PAL25) are presented in Figure 2. Polymerization times were observed at three-minute intervals at room temperature (24 °C). Additionally, optimizing bioink formulations enhances printability while preserving cell viability post printing, which is essential for maintaining tissue functionality.

### 3.2. Physicochemical Analysis

**Figure 3:**
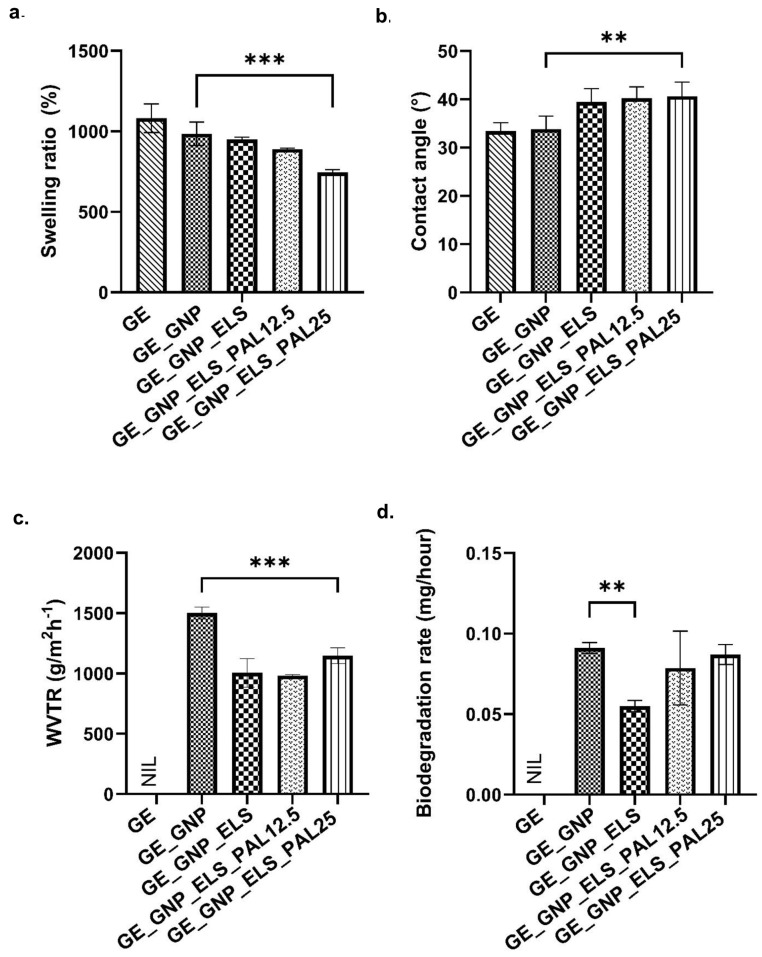
Physicochemical analysis of different hydrogel groups’ (**a**) swelling ratio (%), (**b**) contact angle (°), (**c**) water vapor transmission rate (WVTR) (gm^−2^ h^−1^), and (**d**) biodegradation rate (mg/hour). ** indicates significance at *p* < 0.01 while *** indicates *p* < 0.001.

The physicochemical characterization of gelatin–palmitoyl–GDPH hydrogels was performed through various assessments, including swelling ratio, water vapor transmission rate (WVTR), contact angle, and porosity size. Figure 3 provides quantitative data for each hydrogel group. Hydrogel wound dressings are designed with a high water-absorption capacity to effectively manage the wound exudates. Among the tested groups, non-crosslinked hydrogels (GE) exhibited the highest swelling ratio at 1082.04 ± 72.72%. Gelatin–palmitoyl–GDPH hydrogels, including incorporation with water-soluble elastin (ELS), showed optimal swelling properties exceeding 500%. The results indicate that crosslinked hydrogels (GE_GNP, GE_GNP_ELS, GE_GNP_ELS_PAL12.5, and GE_GNP_ELS_PAL25) retained water in a range from 600% to 900% of their weight. Among the crosslinked formulations, GE_GNP demonstrated the highest water-absorption capacity at 985.00 ± 60.02%, followed by GE_GNP_ELS at 951.00 ± 10.71%. Notably, the swelling ratios of hydrogels containing the palmitoyl–GDPH peptide were lower than GE_GNP. GE_GNP_ELS_PAL25 showed a significantly lower swelling ratio compared to GE_GNP (*p* < 0.001). The swelling ratios for GE_GNP_ELS_PAL12.5 and GE_GNP_ELS_PAL25 were 890.00 ± 5.72% and 746.00 ± 12.96%, respectively.

Figure 3b illustrates contact angle measurements, revealing that all hydrogel groups demonstrated contact angles below 90°, indicative of their hydrophilic properties. The GE hydrogel exhibited the lowest contact angle at 33.44 ± 1.51°, which was not significantly different from the control group (GE_GNP). In contrast, GE_GNP_ELS showed a significantly higher contact angle of 39.46 ± 2.49° compared to GE_GNP. The addition of palmitoyl–GDPH peptide in GE_GNP_ELS_PAL12.5 and GE_GNP_ELS_PAL25 further increased the contact angles to 40.23 ± 2.12° and 40.63 ± 2.67°, respectively, compared to GE_GNP (33.85 ± 2.44°). These results indicate that the treatment groups containing the palmitoyl–GDPH peptide exhibited slightly higher contact angles, reflecting variations in surface hydrophilicity.

The analysis of WVTR presented in Figure 3c demonstrates that crosslinked gelatin–palmitoyl–GDPH hydrogels facilitate water vapor transmission within the acceptable range of less than 1500 gm^−2^ h^−1^, crucial for maintaining an optimal wound environment. The highest WVTR value was observed in GE_GNP at 1503.00 ± 40.50 gm^−2^ h^−1^. Notably, increasing the concentration of palmitoyl–GDPH within the hydrogels enhanced the WVTR, while GE_GNP_ELS_PAL12.5 and GE_GNP_ELS_PAL25 exhibited reduced WVTR values of 983.00 ± 7.35 gm^−2^ h^−1^ and 1148.00 ± 53.15 gm^−2^ h^−1^, respectively, compared to GE_GNP. These findings highlight the role of elastin and palmitoyl–GDPH in modulating WVTR, resulting in significant variations across treatment groups. The biodegradation rate of gelatin–palmitoyl–GDPH hydrogels was quantitatively measured (Figure 3d). The results showed that GE_GNP_ELS exhibited the lowest biodegradation rate (0.055 ± 0.003 mg/h) among the treatment groups, significantly lower than that of the control hydrogel, GE_GNP (0.091 ± 0.003 mg/h). Meanwhile, increasing the palmitoyl–GDPH peptide concentration in GE_GNP_ELS_PAL12.5 and GE_GNP_ELS_PAL25 resulted in higher biodegradation rates of 0.077 ± 0.019 and 0.087 ± 0.005 mg/h, respectively. In contrast, non-crosslinked gelatin (GE) hydrogels degraded completely within an hour of enzyme exposure.

### 3.3. Mechanical Testing Analysis

**Figure 4:**
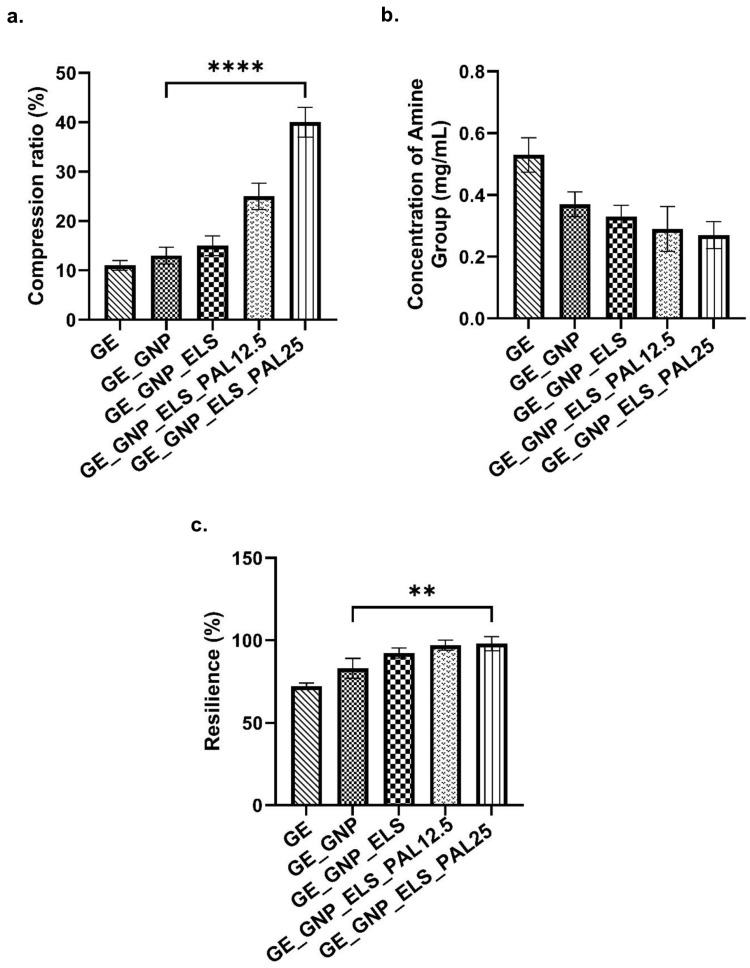
Mechanical properties analysis of different hydrogel groups’ (**a**) % compression ratio, (**b**) concentration of amino group, and (**c**) % of resilience, where ** indicates *p* < 0.01 while **** indicates *p* < 0.0001.

The mechanical strength of biomaterials is critical for their ability to withstand pressure during implantation at wound sites. A compression study was performed to evaluate the mechanical strength of the hydrogels, as illustrated in Figure 4a. Crosslinked hydrogels, including GE_GNP, GE_GNP_ELS, GE_GNP_ELS_PAL12.5, and GE_GNP_ELS_PAL25, exhibited significantly higher compression ratios compared to the non-crosslinked (GE) hydrogel. Among these, GE_GNP_ELS_PAL25 demonstrated the highest compression ratio at 40.00 ± 2.45%, followed by GE_GNP_ELS_PAL12.5 at 25.00 ± 2.16% and GE_GNP_ELS at 15.00 ± 1.63%. These findings emphasize that the incorporation of elastin and palmitoyl–GDPH enhances the mechanical strength of hydrogels. As seen in Figure 4b, all crosslinked hydrogels displayed lower degrees of crosslinking and fewer free amine groups compared to the non-crosslinked GE hydrogel. Additionally, the hydrogels’ resilience was evaluated to determine their ability to recover their shape after pressure or compression. Crosslinked hydrogels containing elastin and palmitoyl–GDPH showed greater resilience than the non-crosslinked GE hydrogel. Therefore, GE_GNP_ELS_PAL25 showed the highest resilience at 98.00 ± 3.56%, significantly outperforming the control group (GE_GNP) at 83.00 ± 4.90%. Figure 4c displays the resilience values for the hydrogels: GE (72.00 ± 1.63%), GE_GNP (83.00 ± 4.90%), GE_GNP_ELS (92.33 ± 2.49%), GE_GNP_ELS_PAL12.5 (97.00 ± 2.45%), and GE_GNP_ELS_PAL25 (98.00 ± 3.56%). These findings highlight the enhanced structural robustness imparted by elastin and palmitoyl–GDPH.

### 3.4. Chemical Characterisation

**Figure 5:**
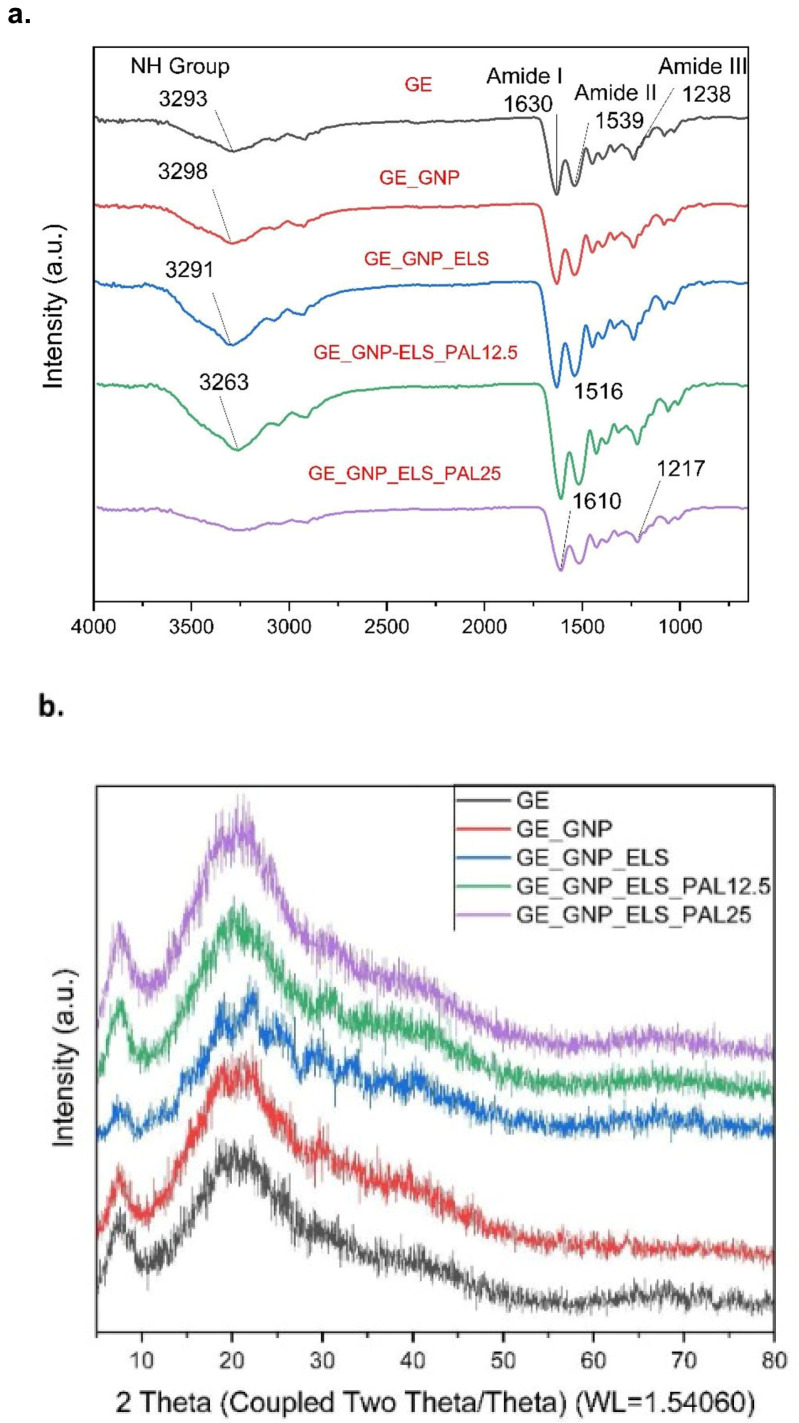
Chemical characterization of different hydrogel groups’ (**a**) FTIR spectra for fabricated hydrogels and (**b**) crystallinity of hydrogels via X-ray diffraction analysis (XRD).

The FTIR spectra in Figure 5a reveal distinct absorption peaks for the various hydrogel groups. In the gelatin (GE) spectrum, a visible peak at 3293 cm^−1^ corresponds to N-H aliphatic and C-H stretching vibrations. Additional peaks at 1539 cm^−1^ and 1630 cm^−1^ are associated with C=O stretching (amide) and N-H bending (amide II), respectively. The GE_GNP_ELS_PAL12.5 hydrogel demonstrated wider peaks at 3293 cm^−1^ and 2989 cm^−1^, which correspond to C=O carbonyl stretching, -CH2 asymmetric stretching, and O-H stretching vibrations of the hydroxy group. An absorption peak at 1630 cm^−1^ was attributed to the stretching vibration of the carboxyl group (C-O), while another peak at 1622 cm^−1^ was detected in both GNP and ELS hydrogels. The FTIR spectra further suggest that the C-H stretching vibration in genipin is responsible for the peak that emerged between 1800 and 3000 cm^−1^.

The XRD profiles of gelatin–palmitoyl–GDPH hydrogels are represented in Figure 5b. XRD is valuable in examining the crystalline phase of scaffolds, providing insights into the mean crystalline size, orientation, and diffraction patterns of gelatin, genipin, elastin, and palmitoyl–GDPH within the structure. A broad peak at 20° in the XRD profile corresponds to the polymer network structure of the hydrogels. The analysis reveals that the addition of genipin, elastin, and palmitoyl–GDPH peptides effectively hinders the crystallization process in the gelatin–palmitoyl–GDPH hydrogels. The crystallinity and amorphous content of hydrogels are summarized in Table 1.

Energy dispersive X-ray (EDX) spectroscopy was used to analyze the elemental compositions of the hydrogels, as shown in Table 2. The presence of carbon (C) was ascribed to the gelatin, water-soluble elastin, and palmitoyl–GDPH peptide components. The XRD data indicated that the incorporation of water-soluble elastin and palmitoyl–GDPH into the gelatin hydrogel matrix led to an increase in in the carbon content within the hydrogels. The GE-GNP hydrogels exhibited a slight decrease in carbon content, accompanied by an increase in oxygen components. However, these changes were not statistically significant.

### 3.5. Three-Dimensional Microporous Structure Hydrogels

**Figure 6:**
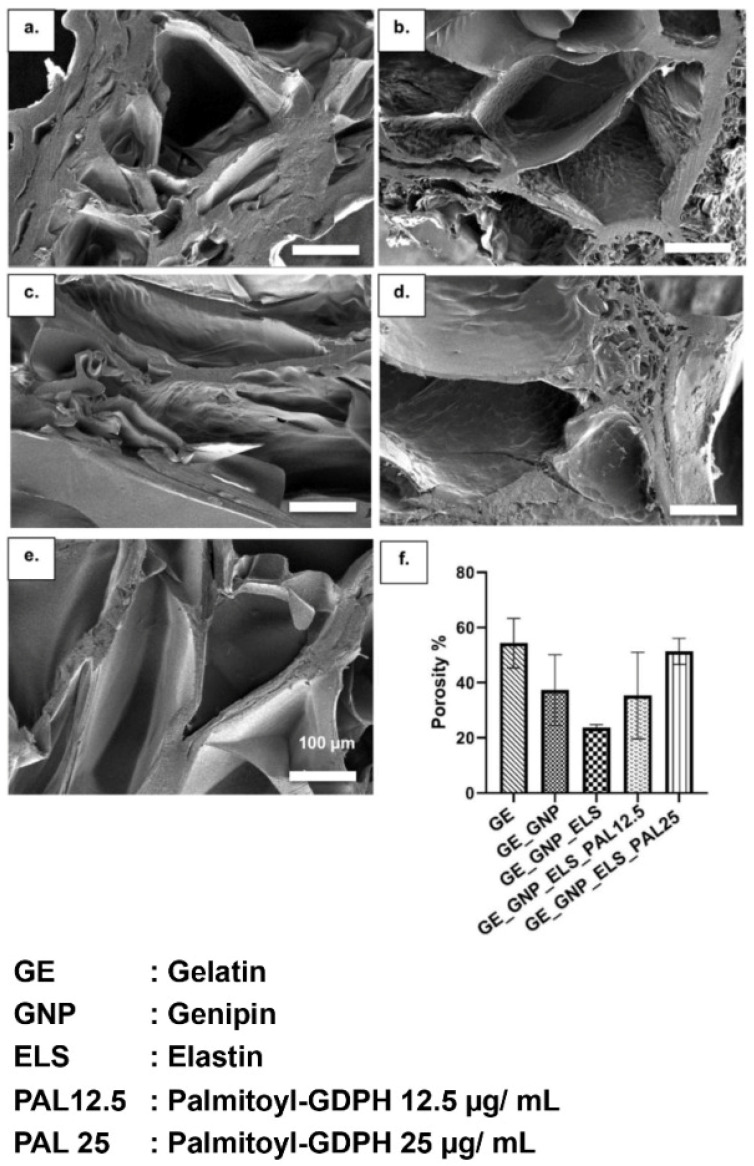
The images of SEM show the cross-sectional microporous structure of the hydrogels: (**a**) GE, (**b**) GE_GNP, (**c**) GE_GNP_ELS, (**d**) GE_GNP_ELS_PAL12.5, and (**e**) GE_GNP_ELS_PAL25 under 100× magnification; (**f**) porosity percentage.

The cross-sectional SEM images in Figure 6a–e show the heterogenic porosity structure of the gelatin–palmitoyl–GDPH hydrogels. Compared to the non-crosslinked GE hydrogel, which showed a more compact microstructure, the crosslinked hydrogels—GE_GNP, GE_GNP_ELS, GE_GNP_ELS_PAL12.5, and GE_GNP_ELS_PAL25—exhibited a well-developed, uniform pore structure with good interconnectivity as a result of the freeze-drying process. Based on the findings, increasing the concentration of palmitoyl–GDPH peptide correspondingly increased the porosity of the hydrogels. This is attributed to the higher concentration of palmitoyl–GDPH in the polymer solution, which enhances the scaffold’s ability to achieve consistent, pore-like structure. Additionally, the microstructure of these hydrogels closely resembles the architecture of natural human skin tissue. Figure 6f presents the porosity percentages of the hydrogels.

### 3.6. Cytotoxicity Assessment

**Figure 7:**
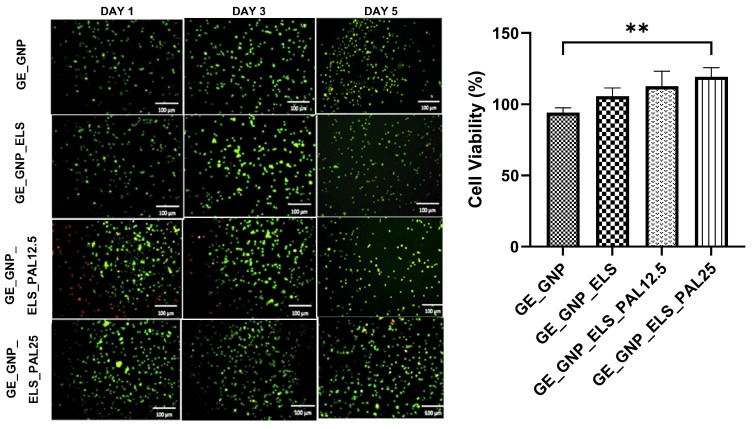
The cytotoxicity of HDFs and their response towards hydrogels were evaluated through live and dead assays and cytotoxic assessments. Cells were seeded and observed in day 1, day 3, and day 5, under 100× magnification and ** indicates *p* < 0.01.

The gelatin–palmitoyl–GDPH hydrogels cultured with HDFs were tested using a live/dead assay, and no cytotoxic effects were observed after 24 h of incubation, as depicted in Figure 7. Green staining indicated live cells, while red staining highlighted dead cells, confirming the biocompatibility and suitability of the hydrogels for cell culture. Overall, among the hydrogel treatment groups, GE_GNP_ELS_PAL25 exhibited the highest percentage of cell viability (119.1 ± 56.86%), followed by GE_GNP_ELS_PAL12.5 (112.5 ± 27.19%), GE_GNP_ELS (105.5 ± 72.39%), and GE_GNP (94.2 ± 46.62%).

### 3.7. Three-Dimensional Bioprinting Assessment

**Figure 8:**
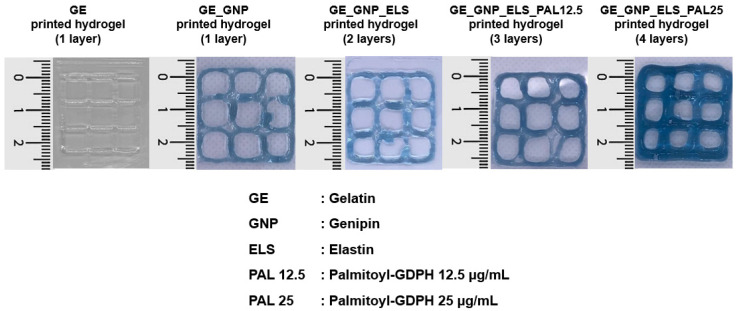
The gross appearance of the 3D-printed hydrogels with different layers.

The potential of the hydrogel formulations as bioinks for future applications in 3D-bioprinting technology, using an extrusion-based approach, was evaluated at room temperature (24 °C). Figure 8 presents the gross appearance of the 3D-printed hydrogels with different layers, including GE, GE_GNP, GE_GNP_ELS, GE_GNP_ELS_PAL12.5, and GE_GNP_ELS_PAL25. The 3D-printed hydrogels were deposited through an extrusion-based bioprinting approach, where material flow was controlled by pressure regulators and speed rates (2000 mm/s), with a constant nozzle diameter of 0.4 mm. Additionally, the 3D-printed hydrogels, which exhibited a blue color, were crosslinked during fabrication before printing.

## 4. Discussion

Advancements in tissue engineering aim to repair damaged tissues that struggle to heal autonomously [44]. Skin tissue engineering is crucial in accelerating the healing process, preventing serious contamination, and also addressing persistent injuries. This study focuses on designing a rapid-gelling injectable hydrogel for cellular treatment and as a bioink for potential future use in 3D-bioprinting applications. The innovative concept behind this biomatrix is its use as a single-application, post-implantation tissue substitute. The integration of cells within the hydrogel is expected to support cellular proliferation, thereby enhancing and expediting the wound-healing process, stimulating cell growth, and effectively speeding up tissue repair and regeneration. Furthermore, scaffolds like hydrogels degrade gradually within the epidermal layer, facilitating tissue regeneration. This research successfully developed hydrogels by combining natural polymers with the synthetic peptide, gelatin, and palmitoyl–GDPH in multiple formulations, presenting a promising therapy for persistent skin injuries. The inclusion of genipin, elastin, and palmitoyl–GDPH was designed to enhance the structural integrity of the hydrogels and promote the wound-healing process by stimulating cell proliferation [28,30,45]. The most optimal formulations were discovered for all hydrogel treatment groups (GE_GNP, GE_GNP_ELS, GE_GNP_ELS_PAL12,5, and GE_GNP_ELS_PAL25), which polymerized in three minutes at room temperature. This three-minute polymerization duration was chosen to provide clinicians sufficient time to apply the hydrogels to the injury site before polymerization [37]. This duration also prevents the hydrogel from fully solidifying, which could complicate extrusion in 3D-bioprinting applications. Also, the presence of genipin increases the ability of hydrogels to polymerize in three minutes by forming covalent bonds with different gelatin amino–polymeric compounds [46].

The physicochemical characteristics were analyzed to further evaluate the performance of the hydrogels. Damaged tissue often experiences significant moisture and water loss compared to intact tissue. Given that conventional wound dressings and artificial tissues often fall short of providing adequate wound drainage, hydrogels emerge as an ideal candidate for skin replacement due to their exceptional capacity to absorb and retain fluids [47,48]. The fabricated gelatin–palmitoyl–GDPH hydrogels exhibit an acceptable swelling ratio, enabling them to effectively absorb excess wound exudate from injury sites. Previous studies suggest that effective hydrogels for wound repair must absorb approximately 500% of their weight to prevent the accumulation of excess wound exudate within the wound region [49,50]. This finding aligns with the properties of gelatin as the primary formulation for this study. However, the addition of water-soluble elastin and palmitoyl–GDPH peptide in the composite scaffolds led to a higher ratio of hydrophilic properties of the hydrogel. This change is attributed to the amino acid composition of water-soluble elastin powder and the use of protease during the extraction process, which builds a hydrophilic protein with an inherent ability to absorb water [30]. Furthermore, the increased concentration of palmitoyl–GDPH results in a higher proportion of water-attracting groups relative to water-repelling ones in the material, which enhances the hydrogel’s compatibility with water molecules. Thus, since GE_GNP_ELS0.2_PAL25 comprises the largest proportion of palmitoyl–GDPH in the hydrogel formulation, the surface contact has the highest contact angle, corresponding to the contact angle tests of fabricated gelatin–palmitoyl–GDPH hydrogels. Moreover, the phenomenon of water contact angle is crucial in defining the characteristics of polymeric matrices and surface morphology, as it significantly influences cell adhesion. This effect is primarily driven by the material’s water solubility and hydrogen bonding capacity, which determine the surface’s hydrophilicity or hydrophobicity [51]. Also, surface roughness is another factor that has been shown to play a significant role in modulating cell behavior, including communication and signaling pathways. Notably, in vitro and in vivo, it directly influences the morphology and proliferation of cells as well as the gene expression patterns of cells [52].

In addition, hydrogels must exhibit an adequate WVTR to maintain an optimal moisture level in the wound environment, which is crucial for effective healing. WVTR characterization is a key factor for wound-healing applications, as it ensures the wound remains hydrated without becoming overly dry or excessively wet. For a hydrogel to serve as an effective skin substitute, the ideal WVTR is typically below 1500 gm^−2^ h^−1^. This range prevents dehydration while supporting the moist conditions necessary to promote cellular migration, proliferation, and tissue regeneration. Thus, the produced gelatin–palmitoyl–GDPH hydrogels exhibit excellent water vapor transmission rate since the outcomes fall between the range of the hydrogels [53]. The hydrogels’ in vitro degradation was another important consideration, as a key limitation of hydrogels is their tendency for quick degradation after the implantation process. So, Figure 3d shows the outcomes of the enzymatic degradation ratio for gelatin–palmitoyl–GDPH hydrogels. Specifically, hydrogels incorporating water-soluble elastin exhibited the prolonged durability than the group of gelatin–genipin. Meanwhile, the presence of palmitoyl–GDPH within hydrogel shows shorter durability. However, for effective wound-healing applications, the degradation duration of the hydrogel at the implanted site should ideally extend to at least 14 days [54]. This timeframe ensures that the hydrogel remains intact long enough to support tissue regeneration and promote optimal healing before it is completely resorbed. The chemical crosslinking process using genipin was also employed to enhance scaffold stability [39]. Genipin not only serves as an effective crosslinker but also offers antioxidant properties, which are beneficial for expediting the wound-healing phases. Additionally, genipin improves the micro-stability of the scaffold, enabling sustained cell migration and differentiation from the surrounding native tissues. Elastin, included as an enhancer in the hydrogel composition, contributes to increased biostability, as corroborated by prior study [55]. The porous, three-dimensional microstructure of the hydrogels allows for efficient passage of water vapor and wound exudates, which is essential to prevent lesion development. It was discovered that all treatment groups of hydrogels exhibited fine walls of pores and uneven architectures of porosity, in contrast to GE_GNP hydrogel. This increase in pore size is attributed to the higher concentration of palmitoyl–GDPH, enhancing the bond between palmitoyl–GDPH and gelatin polymers, in a more loose internal hydrogel structure.

This work effectively developed a gelatin–palmitoyl–GDPH hydrogel that mimics the mechanical characteristics of normal skin, making it an excellent candidate for skin applications. The inclusion of palmitoyl–GDPH significantly enhanced the mechanical properties of the hydrogel compared to the more fragile GE_GNP. Hydrogels with palmitoyl–GDPH demonstrated improved stress and strain resistance, with a compression ratio exceeding 25%, reflecting their robustness under load. This enhancement in mechanical performance is attributed to the functional groups present in the palmitoyl–GDPH peptide, which interact with gelatin molecules, likely forming covalent bonds. These interactions stabilize the hydrogel matrix, resulting in a structure with superior mechanical properties and durability of scaffolds, making it an ideal candidate for supporting tissue repair and regeneration. All hydrogels are intended to be tissue-attached materials that have to adapt to skin’s characteristics as well as resist tearing under strain. The increase in hydrogen bond formation between palmitoyl–GDPH and gelatin contributes to the enhanced stiffness, toughness, and resilience of the hydrogels. This interaction results in stronger material properties, making them more suitable for dynamic applications. Thermodynamically, the elastic polymer network within these composite hydrogels provides exceptional resilience due to their supramolecular nature. Figure 4c highlights that GE_GNP_ELS_PAL12 and GE_GNP_ELS_PAL25 exhibit significantly greater resilience compared to crosslinked gelatin-only (GE_GNP) hydrogels. Prior research supports this observation, showing that an increase in hydrogen bond interactions between palmitoyl–GDPH and gelatin improves the hydrogels’ mechanical properties, including their stiffness, toughness, and resilience, all improvements underscoring the potential of these hydrogels as effective skin substitutes capable of maintaining mechanical integrity while adhering to and mimicking native skin properties.

The non-crosslinked gelatin exhibited characteristic peaks of amide I and amide II, as shown in Figure 5a. These were attributed to N-H stretching vibrations (amide I) and aliphatic C-H stretching vibrations (amide II), respectively, and significant changes were observed at the amide II peak after the crosslinking application, confirming that the triple-helix structure of gelatin was preserved. Furthermore, the XRD characterization results show the percentage of crystallinity. The crystallinity of the hydrogel samples was analyzed using XRD, as shown in Figure 5b. For all hydrogel samples, the XRD patterns revealed a consistent pattern of broader peak in the 10–30° (2θ) range, indicative of an amorphous structure. The absence of new peaks in the XRD patterns for the composite hydrogels confirmed the compatibility of palmitoyl–GDPH with gelatin. This also indicated that no new phase was introduced within the synthesized polymeric matrix system. These observations confirm that the inherent characteristics of the hybrid biomaterials were preserved despite the introduction of the natural crosslinking agent genipin and synthetic peptide palmitoyl–GDPH. Maintaining the native properties of the materials is critical to ensuring that the targeted cells interact optimally, promoting their performance while minimizing cell death. This balance is essential for the hydrogels’ effectiveness as biomaterials in wound-healing applications.

Cell viability assessments are a critical component of toxicity testing, providing insights into cellular responses to potential toxicants or novel substances. In this study, no evidence of cell membrane damage was observed after treatment with palmitoyl–GDPH at concentrations of 12.5 µg and 25 µg, as demonstrated in Figure 7. The results clearly showed that cell viability on GE_GNP_ELS_PAL12.5 and GE_GNP_ELS_PAL25 hydrogels was comparable to the positive control, with no significant toxicity detected. These findings affirm that the optimized incorporation of palmitoyl–GDPH into gelatin hydrogels promotes cell adhesion and viability, enhancing the scaffold’s biocompatibility. This observation is consistent with a previous study by Fadilah et al. [28], which demonstrated that human dermal fibroblasts (HDFs) remained viable after exposure to various concentrations of palmitoyl–GDPH. The enhanced biocompatibility of the hydrogels suggests their potential for supporting cellular processes essential for tissue regeneration and wound healing.

The primary goal of this study was to characterize the composition of hydrogel with an injectable approach to propose a possible bioink in the application of bioprinting with the 3D bioprinter. Hence, the initial finding was obtained by optimizing the overall visual appearance of the hydrogels, as shown in Figure 8. The findings reveal that increased levels of palmitoyl–GDPH were effectively developed at ambient temperature owing to its elevated viscosity and rapid transformation from a semi-solid to a fully solid phase, which are critical factors for 3D bioprinting, as this property directly impacts the integrity and standard of scaffolds. However, one limitation of gelatin-based hydrogels is the reversible nature of their sol–gel transition, which can lead to degradation over a few hours when incubated in growth media at 37 °C. To address the limitations, the commercial GE solutions were blended with water-soluble elastin and palmitoyl–GDPH and then chemically linked through crosslinking using genipin. This combination enhanced the structural stability of the hydrogel, preventing its melting under physiological conditions. The addition of palmitoyl–GDPH also optimized the printing temperature to 23 ± 2 °C at room temperature, as illustrated in Figure 8, ensuring the production of high-quality printed scaffolds. Furthermore, the bioink printing temperature can be finely regulated and kept steady with the help of an extruder and a controlled printing surface. These capabilities highlight hydrogel’s potential as a robust bioink for precise and efficient 3D-bioprinting applications. Nevertheless, more in vitro and in vivo studies need to be conducted later to enhance the assessment of the produced gelatin–palmitoyl–GDPH hydrogels’ effectiveness as functional biomaterials (injectable method) and promising bioinks (3D bioprinting) for the treatment of wounds.

## 5. Conclusions

Overall, the successful fabrication and polymerization of an injectable crosslinked gelatin–palmitoyl–GDPH hydrogel in a duration of three minutes at room temperature (22–24 °C) implies its promise for use in conventional methods such as injectable hydrogel and advanced technology of 3D bioprinting with bioinks. Water-soluble elastin, palmitoyl–GDPH, and genipin were incorporated into the gelatin hydrogels to increase their mechanical characteristics and enhance their in vitro physicochemical and biological evaluation as a material for wound dressings. Biomimetic gelatin–palmitoyl–GPH hydrogels were found to be promising biomaterials with increased hydrophilicity and capacity for water absorption based on their ability to absorb wound exudate and their appropriate swelling properties (>500%), allowing the hydrogels to create a moist wound environment. These properties could be useful in applications like chronic wound-healing treatment. It was discovered that the hydrogels were biocompatible with HDFs as a therapeutic for cellular wound repair. The effectiveness of the hydrogel formulations, which may enhance cellular interaction at the implanted site, was demonstrated by the live/dead assay and cell viability, which was >100%. Nevertheless, there are several limitations to this study. As the presented study mostly concentrated on in vitro assessments, although the findings are promising, further in vivo investigations are required to verify the biocompatibility, safety, and wound-healing efficacy of the hydrogels in a physiological setting. Furthermore, the hydrogels’ stability and therapeutic efficacy may be jeopardized by the possibility of enzymatic degradation in vivo, which calls for additional research to optimize their rates of degradation. The long-term consequences of hydrogels are yet unknown, especially how well they work in models of chronic wound healing, which remain unexplored. Also, since this gelatin-based hydrogel is incorporated with the peptide, the scalability and expense of therapeutic agents of the peptide itself provide significant obstacles to their wider application. Peptide synthesis is frequently costly and requires intricate processes, which makes large-scale manufacture difficult. These limitations might prevent gelatin–palmitoyl–GDPH hydrogels from being widely used therapeutically. Thus, addressing these issues is vital to unlocking the full potential of peptide-incorporated gelatin-based hydrogels for chronic wound-healing applications.

## Figures and Tables

**Table 1:** Crystallinity and amorphous content in GE_GNP_ELS_PAL hydrogels as determined by XRD analysis.

Group of Hydrogels	Crystallinity (%)	Amorphous (%)
GE	42.8%	57.2%
GE_GNP	48.8%	51.2%
GE_GNP_ELS	25.8%	74.2%
GE_GNP_ELS_PAL12.5	20.0%	80.0%
GE_GNP_ELS_PAL25	18.3%	81.7%

**Table 2:** Elemental composition of the hydrogels as determined by EDX. The hydrogel groups showed varying levels of oxygen, carbon, and nitrogen.

Hydrogels	C (%)	O (%)	Na (%)
GE	61.0 ± 0.35	19.3 ± 0.22	-
GE_GNP	60.9 ± 0.35	23.4 ± 0.21	-
GE_GNP_ELS	61.5 ± 0.90	33.6 ± 0.90	4.9 ± 0.30
GE_GNP_ELS_PAL12.5	67.5 ± 1.00	31.8 ± 1.00	0.8 ± 0.20
GE_GNP_ELS_PAL25	65.7 ± 1.50	33.7 ± 1.50	0.6 ± 0.20

## Data Availability

The original contributions presented in this study are included in the article. Further inquiries can be directed to the corresponding author.
